# Supplementary material for: Identification and detection of genetic markers associated with antimicrobial susceptibility and evaluation of efflux pump mechanisms in Mycoplasma iowae
Source: PLoS One. 2026 Apr 17;21(4):e0347345. doi: 10.1371/journal.pone.0347345 (PMC13089696; doi:10.1371/journal.pone.0347345)
Supplement: Attachment — Submitted filename: Response_to_Reviewers.docx [file pone.0347345.s008.docx]

Zsuzsa Kreizinger

HUN-REN Veterinary Medical Research Institute

Hungária krt. 21.

H-1143 Budapest, Hungary

Phone: +36 1 467 4060 Fax: +36 1 467 4076
E-mail: kreizinger.zsuzsa@vmri.hun-ren.hu

Chih-Horng Kuo

Academic Editor

PLOS One

27^th^ February, 2026

Dear Chih-Horng Kuo,

We thank You and the Reviewer for Your e-mail on 3^rd^ of January, 2026 and for Your careful evaluation of our manuscript” Identification and detection of genetic markers associated with antimicrobial susceptibility and evaluation of efflux pump mechanisms in *Mycoplasma iowae*” and for the constructive comments provided. The suggestions have significantly improved the clarity, and overall quality of the manuscript.

**I. Response to Journal Requirements**

**1. Compliance with style requirements and file naming**

We have thoroughly reviewed the manuscript to ensure full compliance with the journal’s style and formatting requirements, including correct file naming conventions. Hopefully, all structural, formatting, and stylistic requirements have now been met.

**2. Code sharing requirements**

We fully agree with the journal’s policy regarding code transparency and reproducibility. In the revised version of the manuscript programs (Geneious, Promotech) were used according to the best practice that their developers recommend for sequence analyses, and online tools (Statistics Kingdom, <https://www.socscistatistics.com/>) were applied to perform the statistical analyses (logistic regression, Fisher’s exact test, Wilcoxon Signed-Rank test), hence the codes are available at the corresponding references. Mutations were further subjected to manual review and categorization, without the use of any codes. The text has been modified (Lines 140-157, 240-245, 276-281, 284-291, 294-297, 386), and data used for the statistical analyses and the corresponding results are now available in Tables S3-S6.

**3. Funding statement clarification**

Thank you for this important clarification. We have now explicitly included the following statement in our Funding statement and cover letter:

“The funders had no role in study design, data collection and analysis, decision to publish, or preparation of the manuscript.”

**4. Funding information placement**

We acknowledge the requirement that funding information must appear exclusively in the Funding Statement section of the online submission form. Accordingly, all funding-related text has been removed from the Acknowledgments. The amended Funding Statement has been included in the cover letter as requested. We respectfully request that the online submission form be updated accordingly.

**5. “Data not shown” statement**

We acknowledge that the phrase “data not shown” does not meet data sharing standards. All relevant data previously referred to as “data not shown” have now been included in the manuscript (Lines 357-358, 386-389, 508-509).

**6. Citation of reviewer-recommended publications**

We have carefully evaluated all suggested references. Relevant publications have been incorporated into the revised manuscript where appropriate (statistical analysis: citations No. 36-38; effect of reserpine in *Mycoplasma pneumoniae*: No. 54). In order to clarify nucleotide positions, the numbering has been given not only according to *Escherichia coli*, but according to the type strain of *Mycoplasma iowae* too, as recommended in the review of molecular assays for AMR detection by the MyMIC Consortium (citation No. 39; Lines 135-137, 159-160; Tables S1).

**II. Additional Editor Comments**

**1. Clarification of resistance mechanisms and discussion of unexplained phenotypes**

We have clarified in the Materials and Methods and Results sections which resistance mechanisms were examined, including SNP analyses and gene screening. Additionally, we have expanded the Discussion section to explicitly address resistant phenotypes that remain unexplained and have framed these as study limitations. We hope, that the need for further investigation of alternative mechanisms is now clearly acknowledged.

**2. Statistical support for SNP–MIC associations**

We agree that statistical support strengthens genotype–phenotype interpretation, even if the study first evaluated the nucleotide positions based on literature data about their previous association to resistance. The recommended statistical analyses (including Fisher’s exact test and logistic regression) have now been performed to evaluate SNP–MIC associations. The Results and Materials and Methods sections have been updated accordingly.

**3. Moderation of conclusions regarding efflux pump involvement**

The conclusions regarding efflux pump involvement have been carefully moderated. We now clearly state the limitations of inhibitor-based assays and avoid overinterpretation of efflux activity based solely on MIC reduction. The Discussion section has been revised to reflect a more cautious and evidence-based interpretation.

**4. Alignment of novelty and diagnostic applicability claims**

We have carefully revised statements regarding novelty and diagnostic applicability to ensure that they accurately reflect the scope and strength of the data. Claims have been moderated and better aligned with the evidence presented.

**III. Reviewer’s Concerns**

**Major Concerns**

**1: Incomplete results of resistance mechanisms**

We agree that a broader investigation of resistance mechanisms would strengthen the manuscript. However, as - to our knowledge – a first comprehensive investigation in this specific field, our intentions were to assess the presence of known mutations for target modification and develop diagnostic assays for their differentiation. However, *Mycoplasma iowae* proved to have very unique characteristics among the avian pathogen mycoplasmas, and in the absence of official standards in MIC testing in mycoplasmas, and the detected difference in MIC values in the different media, the description of new associations was beyond the scope of our aims. Thus, we completed our study with the inhibitor-based assay to assess the possibility of involvement of an efflux pump activity in the development of resistance.

Thank You for pointing out the search for tetracycline resistance-associated mutations without reported results. Since the examined strains showed only slightly elevated MIC values - equal or below 4 µg/ml in Oxoid media (the MIC values which were used for the association study) - they were all considered susceptible to oxytetracycline, therefore genetic markers were not searched in the corresponding regions, and accordingly the sentence has been deleted from the Materials and Methods (Lines 128-129, 139-140). For macrolide resistance, results of the analysis of ribosomal proteins (L4 and L22, corrected as appropriate) have now been included (Lines 298-300).

We agree that the findings of four isolates with elevated MICs lacking 23S rRNA position 2059 alterations warrant further investigations, which we now highlight in the discussion (Lines 454-458). As two of these strains were involved in the inhibitor-based assays in case of tilmicosin, we also discuss their phenotype changes caused by orthovanadate in Lines 522-526.

**2: Lack of statistical support for genotype–phenotype associations**

Following the Reviewer’s recommendations, statistical analyses (Fisher’s exact test and logistic regression) comparing MIC distributions between wild-type and mutant genotypes for *gyrA*, *parC*, and 23S rRNA have now been conducted. Statistical methods and results are described in the Materials and Methods and Results sections (Lines 140-157, 276-281, 284-291, 294-297), and data used for the statistical analyses and the corresponding results in details are now available in Tables S3-S6.

**3: Limited efflux pump functional validation**

We appreciate the valuable suggestions regarding the functional validation of efflux pump activity. Although we are not in the position to perform further experiments, such as the continuation of the studies with reserpine, and transcriptional or protein-level validation was beyond the scope of the current study, we now clearly acknowledge these limitations in the Discussion, and indicate them as important directions for future research. We have also explicitly discussed the limitations of inhibitor-based validation. (Lines 512-518, 528-530)

**Minor Comments**

**Ad 1.**

The Abstract has been shortened and streamlined (Lines 35-55). It now clearly states that MAMAs were developed specifically for fluoroquinolone resistance-associated SNPs (Lines 42-44, 53-54).

**Ad 2.**

The phrase “and were aligned” has been deleted as suggested (Line 262).

**Ad 3.**

In accordance with the Reviewer’s correction, the discrepancy between the text and Table 2 has been arranged. Primer specificities (including clarification that no allele-specific primer was designed for 248A) are now accurately described and results for the non-specific alleles are presented (Lines 320-329).

**Ad 4.**

We agree that the interpretation of mixed genotypes is an important practical limitation. The potential for false-negative detection of *parC* H genotype in mixed populations has now been explicitly discussed in the Discussion section (Lines 488-495).

**Ad 5.**

Figure 2 has been revised to include all six antimicrobials to provide a comprehensive overview. The figure now shows the log2 transformed MIC values of the antibiotics in Oxoid media, MolliScience, and MolliScience supplemented with sodium orthovanadate. The log2 transformed MIC values of the two-fold dilution series of antimicrobial concentrations are shown to normalize data distribution.

**Ad 6.**

Table S2 has been updated to include the genomic positions of the efflux pump genes according to the type strain of *Mycoplasma iowae*, and the text has been completed regarding how regions were analyzed and positions were identified (Lines 263-265). In addition, in order to clarify nucleotide positions in the whole manuscript, the numbering has been given not only according to *Escherichia coli*, but according to the type strain of *Mycoplasma iowae* too, as recommended in the review of molecular assays for AMR detection by the MyMIC Consortium (citation No. 39; Lines 135-137, 159-160, Tables S1).

We sincerely thank the Editor and Reviewer for their constructive and thoughtful evaluation. We believe that the revisions have significantly strengthened the scientific accuracy, clarity, and transparency of the manuscript.

Sincerely,

Zsuzsa Kreizinger
On behalf of all co-authors
