# Supplementary material for: Identification and detection of genetic markers associated with antimicrobial susceptibility and evaluation of efflux pump mechanisms in Mycoplasma iowae
Source: PLoS One. 2026 Apr 17;21(4):e0347345. doi: 10.1371/journal.pone.0347345 (PMC13089696; doi:10.1371/journal.pone.0347345)
Supplement: Attachment — Submitted filename: Response_to_Reviewers_auresp_2.docx [file pone.0347345.s009.docx]

Zsuzsa Kreizinger

HUN-REN Veterinary Medical Research Institute

Hungária krt. 21.

H-1143 Budapest, Hungary

Phone: +36 1 467 4060 Fax: +36 1 467 4076
E-mail: kreizinger.zsuzsa@vmri.hun-ren.hu

Chih-Horng Kuo

Academic Editor

PLOS One

30^th^ March, 2026

Dear Chih-Horng Kuo,

We thank You and the Reviewer for Your e-mail on 17^th^ of March, 2026 and for Your careful evaluation of our manuscript” Identification and detection of genetic markers associated with antimicrobial susceptibility and evaluation of efflux pump mechanisms in *Mycoplasma iowae*” and for the constructive comments provided.

**I. Response to Journal Requirements**

**Citations**

We have carefully evaluated all suggested references during the previous revision, no new recommendations were added. Relevant publications have been incorporated into the revised manuscript where appropriate (statistical analysis: citations No. 36-38; effect of reserpine in *Mycoplasma pneumoniae*: No. 54). In order to clarify nucleotide positions, the numbering has been given not only according to *Escherichia coli*, but according to the type strain of *Mycoplasma iowae* too, as recommended in the review of molecular assays for AMR detection by the MyMIC Consortium (citation No. 39).

All statistical analyses were repeated or performed according to the first revision of the manuscript using the cited tools, therefore the previously cited software (R 4.3.3) has been eliminated from the citation list.

A full audit with publisher-level verification was conducted using ChatGPT to assess the reference list for retractions. This included checking direct sources (PubMed and official publisher pages) for retraction status, reviewing PubMed retraction flags, examining publisher platforms (Elsevier, Taylor & Francis, Springer, Frontiers, PLoS), and performing Retraction Watch–type searches using title and author combinations. No retracted publications or linked retraction notices were identified in our reference list.

**II. Reviewer’s Comments**

**Ad 1.**

Figure 2 has been adjusted and now split axis is used instead of log2MIC bars to better visualize MIC values, the description of the figure has been corrected accordingly (Fig2, Lines 392, 396-397).

**Ad 2.**

In accordance with the Reviewer’s comment, we completed our discussion about unexplained resistant phenotypes as limitations with the unexplained susceptible phenotype in case of macrolides and lincomycin (Lines 455-457).

**Ad 3.**

According to the clarified MAMA specificity, the text has been corrected when the MAMA-targeted mutation was mentioned (Lines 322, Table 3). The discussion details examinations on the sequences and MIC values, not on the MAMA results, therefore in these cases, the “C248T/A” form was considered appropriate (Lines 469, 481). Nevertheless, the specificity of the gyrA-MAMA has been addressed in the discussion also (Lines 484-485).

We thank the Editor and Reviewer for their evaluation. We consider that the revisions have improved the scientific accuracy, clarity, and transparency of the manuscript.

Sincerely,

Zsuzsa Kreizinger
On behalf of all co-authors
